# Effect of long-term GH treatment in a patient with CHARGE association

**DOI:** 10.1186/1824-7288-40-51

**Published:** 2014-06-02

**Authors:** Andrea Esposito, Maria Tufano, Iolanda Di Donato, Martina Rezzuto, Nicola Improda, Daniela Melis, Mariacarolina Salerno

**Affiliations:** 1 Pediatric Section, Department of Medical Translational Sciences, University “Federico II” of Naples, Naples, Italy

**Keywords:** CHARGE association, GH deficiency, GH treatment, Adult height

## Abstract

CHARGE association is characterized by ocular Coloboma, Heart malformations, choanal Atresia, Retardation of growth and development, Genital abnormalities and inner and external Ear abnormalities. Growth failure is a frequent find mainly associated with feeding difficulties or systemic diseases. To date, GH deficiency has been reported in only few patients with CHARGE association however long-term effects of GH treatment, up to final height, have never been reported. We describe a patient with CHARGE association and GH deficiency treated with GH from the age of 3 years and 10 months up to adult height.

## Background

CHARGE association is a multiple-malformation syndrome that includes ocular Coloboma, Heart malformations, choanal Atresia, Retardation of growth and development, Genital abnormalities and inner and external Ear abnormalities [1]. The majority of subjects with CHARGE association presents mutations involving the chromodomain helicase DNA-binding protein-7 (*CHD7*) gene on chromosome 8q12 [2].

Developmental delay and growth retardation become more evident with age. At birth, children with CHARGE association usually have normal weights and lengths and the most marked decline away from the centiles is observed during infancy with a significant limited catch-up growth in stature [3]. Although anterior pituitary dysfunction has not always been identified as the cause of growth failure in patients with CHARGE association, GH deficiency (GHD) has been reported in few cases [3-5]. To our knowledge, only two patients with CHARGE association and GH deficiency have been treated with recombinant GH therapy [6] but the impact of long-term therapy has not been reported so far.

Hereby we report a case of a patient with CHARGE association and GHD treated with GH from the age of 3 years and 10 months up to final height.

## Case presentation

The patient was a boy born by cesarean delivery at 40 weeks of gestation following a pregnancy complicated by polidramnios. He was the first of three children born to healthy, non-consanguineous parents. His birth weight was 2.800 kg (-1.6 SDS) with an Apgar score of 5 at 1 minute and 6 at 5 minutes. At birth he was admitted to Neonatal Intensive Care Unit with respiratory distress and underwent surgical treatment for esophageal atresia. At physical examination he showed dismorphic facial features (external ear sloping forehead, flattened tip of nose, left microphtalmia), nistagmus and general hypotonia. The ophtamological inspection showed coloboma of retina involving the optic nerve. Visual evoked response showed immature waves. Diagnostic exams aimed at recognizing malformations were performed: abdomen ultrasound was normal and renal anomalies were not detected; echocardiogram revealed aortic arch ectasia and aortic valve regurgitation.

At 12 months of life speech delay was noted and auditory brainstem response showed mixed deafness. Hearing aid device and logopedic rehabilitation were required.

At the age of 14 months the patient developed gastroesophal reflux, that required medical treatment. Psychomotor delay required cognitive therapy since first years of life. Throughout childhood his language and social skills progressed but remained moderately delayed. Challenging behaviors including agitation, depression, anxiety and problem with selfregulation required behavior therapy.

Based on these clinical features, CHARGE association was suspected and genetic investigations were performed. Karyotype was normal and excluded 22q11 deletion as well as abnormalities of chromosomes 22, 14, and 9; fluorescent in situ hybridization excluded 22q11 deletion; *CHD7* gene mutation testing showed heterozygotic mutation c. 4789_4790 of the exon 21.

Because of severe growth impairment by the age of 3 years and 10 months he underwent endocrine evaluation. Height was 88.3 cm (-2.95 SDS), quite below the target height (TH) (170.5 cm, -0.96 SDS), BMI SDS was -0.86, growth velocity was 4.8 cm/year (-2.09 SDS) [7] and bone age was more than 2 years delayed with respect to chronological age.

Malnutrition and systemic causes of short stature were excluded, thyroid function was normal while IGF-1 concentration was low (28 ng/ml, -1.17 SD).

GH secretion, evaluated after two different pharmacological tests, showed a mild GH deficiency (GH peak after L-DOPA 6.0 ng/ml and GH peak after arginine 6.8 ng/ml respectively) [8]. Hypothalamus-pituitary region magnetic resonance imaging showed partial empty sella.

**Figure 1:**
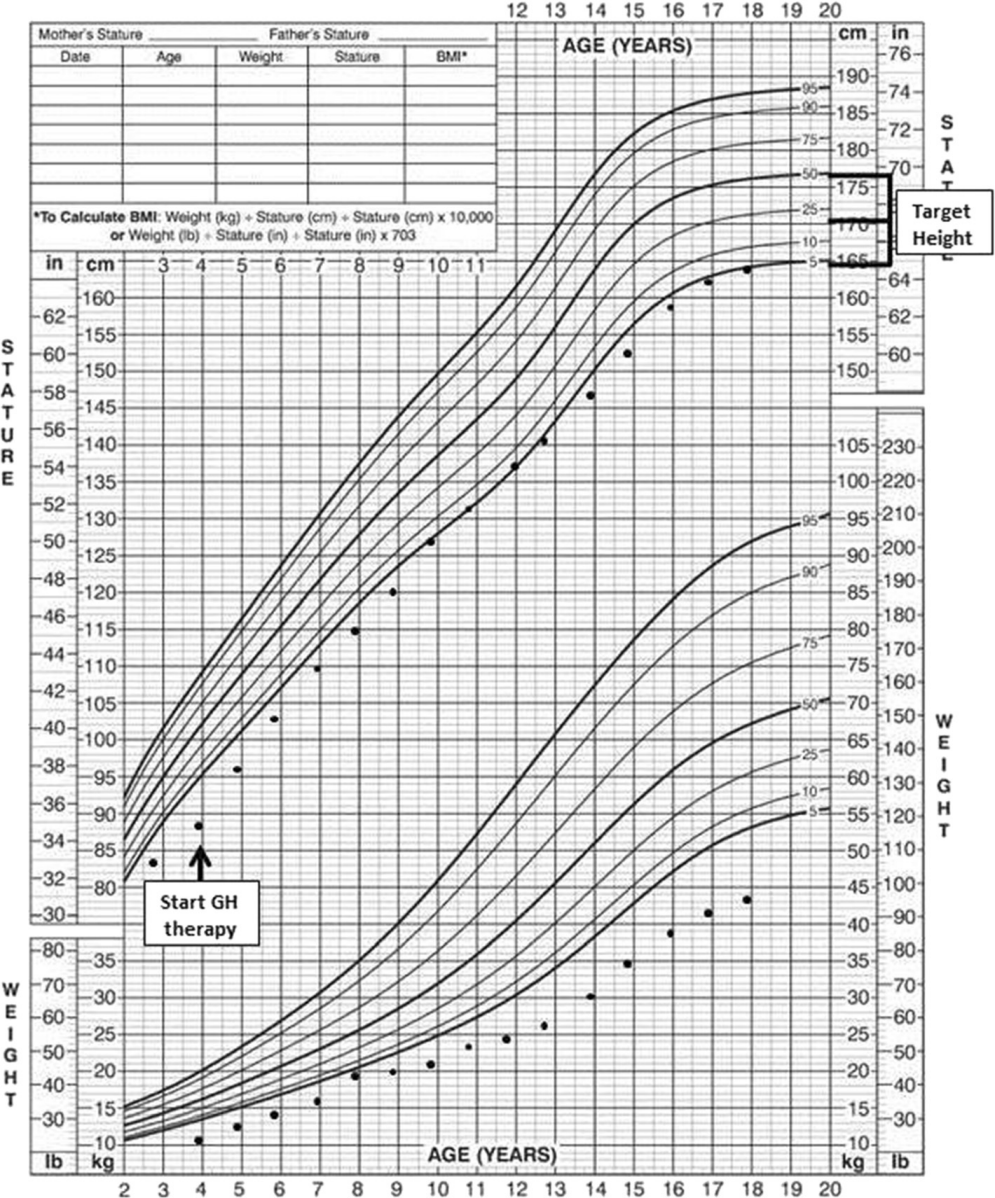
Linear growth of the patient before and during GH therapy up to adult height.

Therapy with recombinant GH was initiated at the age of 3 years and 10 months at the dosage of 30 μg/kg/die with a consequent increase in growth velocity to 7.7 cm/year (0.77 SDS). No additional hormone deficiencies were observed during follow-up. A mild increase in serum TSH levels (TSH 5.0 mU/l) with normal FT4 was observed during the last two years of GH treatment; however that did not required any therapy [9]. Wechsler Intelligence Scale for Children performed at the age of 10 years confirmed mild developmental delay (Intelligence Quotient 67). Pubertal development began spontaneously at the age of 13 years and 4 months and progressed regularly. No GH-related adverse events occurred during the follow-up.At the age of 17 years and 10 months GH treatment was stopped. His adult height was 164 cm (-1.80 SDS), in the low range of his TH (Figure 1).

## Discussion

CHARGE association is a well-established multiple-malformation syndrome that includes Coloboma, Heart defect, Atresia choanae, Retarded growth and development, Genital hypoplasia, Ear anomalies/deafness [1].

A diagnosis of CHARGE association should be considered in any infant with coloboma, choanal atresia, asymmetric facial palsy or classical CHARGE ears in combination with other specific congenital anomalies [1]. Individuals with all four major characteristics (the classical 4C’s: Choanal atresia, Coloboma, Characteristic ears and Cranial nerve anomalies) or three major and three minor characteristics are highly likely to have CHARGE association [1,10].

*CHD7* has been reported to be a causative gene of CHARGE association and mutations in this gene have been reported in the majority of patients with CHARGE association [11]. In situ hybridization analysis of the *CHD7* gene during early human development showed a good correlation between CHD7 expression patterns and the developmental anomalies observed in CHARGE association [12].

CHD7 expression has been reported within the hypothalamus and pituitary gland suggesting that endocrine deficiencies may occur in CHARGE association as consequence of the differentiation of either the hypothalamic nuclei or the trophic cells of the anterior pituitary [5,12]. Indeed, CHD7 has been described to be a SOX2 cofactor which is an important regulator of hypothalamic-pituitary axis [13]. In fact, structural pituitary abnormalities as anterior pituitary hypoplasia and ectopic posterior pituitary have been reported in patients with CHARGE association [6,14].

Hypogonadotropic hypogonadism (HH) is the most frequent endocrine feature described in CHARGE association [5] whilst GH, TSH and ACTH deficiencies have been occasionally reported [6].

In some cases HH may be responsible for the hypogenitalism which is considered a major feature of CHARGE association presenting in males with micropenis and/or cryptorchidism in infancy while females usually do not show evidence of genital hypoplasia [15]. Conversely, delayed puberty and biochemical findings of HH (FSH and LH low or undetectable) have been reported in both male and female subjects [4-6,14,15]. In our patient, however, HH was excluded by the presence of normal genitalia in infancy (penis length 4.7 cm with normal testes in the scrotum at the age of 3 years and 10 months) and by spontaneous pubertal development during adolescence.

Moreover, growth retardation is an hallmark of CHARGE association. Most children have normal length at birth [3] however, suboptimal postnatal linear growth may occur in up to 90% of subjects, especially in the first three years of life [4]. Therefore, mean adult height is usually at or below the 3rd percentile [4,10] even if the attainment of normal adult height has been occasionally reported [16]. In CHARGE association growth failure is frequently due to feeding difficulties or renal, gastrointestinal, cardiovascular anomalies. However, few patients with CHARGE association have been diagnosed to have GH deficiency [5,6,13]. GH-IGF1 axis defects have been reported in other genetic syndromes. However, GH treatment is not always associated with optimal results in term of linear growth [17]. Only two other patients with CHARGE association have been so far treated with GH [6]. They presented multiple pituitary hormone deficiencies with small anterior pituitary and ectopic posterior pituitary. Short-term GH therapy was associated with a significant increase in growth velocity; however, in these patients long-tem effects of GH-treatment are not yet appreciable as they have not yet reached puberty and final height.

To our knowledge, it is the first report of long-term (14 years) GH treatment in a patient with CHARGE association. In our patient GH deficiency was documented after the exclusion of other systemic causes of short stature. Moreover, the radiological finding of partial empty sella was consistent with GHD diagnosis. GH treatment was associated with a great improvement in growth rate and resulted in a final height appropriate to his genetic target without any adverse event.

## Conclusions

Our report confirms that growth retardation in CHARGE association can be due to GH deficiency and that in these patients long-term GH treatment can have a significant positive effect on growth velocity and adult height. Therefore, biochemical evaluation of GH-IGF1 axis should be performed in children with CHARGE association and growth retardation after exclusion of malnutrition and other causes of systemic diseases.

## Consent

Written informed consent was obtained from the patient’s parents for publication of this Case Report and any accompanying images.

## Abbreviations

CHD7: Chromodomain helicase DNA-binding protein-7; GHD: Growth hormone deficiency; TH: Target height; HH: Hypogonadotropic hypogonadism.

## Competing interests

The authors declare that they have no competing interests.

## Authors’ contributions

All authors have participated in drafting of the manuscript and/or critical revision of the manuscript for important intellectual content. All authors read and approved the final manuscript.
